# Workplace Barriers to Nursing Autonomy in Saudi Hospitals: Implications for Management and Policy Under Vision 2030

**DOI:** 10.1155/jonm/6154169

**Published:** 2026-03-28

**Authors:** Ibrahim Naif Alenezi

**Affiliations:** ^1^ Public Health Nursing Department, College of Nursing, Northern Border University, Arar, Saudi Arabia, nbu.edu.sa

**Keywords:** healthcare reform, interprofessional collaboration, nursing autonomy, qualitative research, Saudi Arabia, Vision 2030

## Abstract

**Aim:**

This study explored how nurses perceive and navigate professional autonomy in Saudi hospital settings, with particular attention to the organisational and cultural barriers that shape autonomous practice.

**Background:**

Vision 2030 provides the strategic framework for transforming healthcare systems in Saudi Arabia, placing nursing autonomy at the centre of efforts to deliver safe, patient‐centred care. Nursing autonomy influences clinical decision‐making, job satisfaction and workforce retention.

**Methods:**

A qualitative descriptive design was employed across two referral hospitals in northern Saudi Arabia. Purposive sampling was used to recruit staff nurses and nurse managers with direct experience of clinical autonomy in practice. Twenty‐four semistructured interviews were conducted in Arabic with staff nurses (*n* = 16) and nurse managers (*n* = 8). Data were analysed using Braun and Clarke’s reflexive thematic analysis.

**Results:**

Three themes were constructed through analysis: (1) structured dependency, reflecting how hierarchical systems restrict independent decision‐making; (2) navigating interprofessional dynamics, addressing collaborative practice and role negotiation and (3) communication and professional identity, capturing how nurses’ sense of agency is shaped by confidence, visibility and perceived professional status.

**Discussion:**

The findings indicate that nursing autonomy is variably supported and is shaped by the interplay of hierarchical structures, interprofessional dynamics and communication‐based professional identity work.

**Conclusions:**

A substantive misalignment exists between the strategic aims of Vision 2030 and nurses’ day‐to‐day experiences of hierarchical control and structured dependency. Addressing this gap requires targeted interventions, including leadership development and sustained institutional commitment to shared governance models.

## 1. Introduction

Nursing autonomy is an important principle in modern healthcare practice, enabling nurses to make independent, professional decisions in clinical practice [1–3]. It encourages interprofessional collaboration, improves patient care outcomes and raises nurses’ status within healthcare systems [2, 4]. Healthcare in Saudi Arabia is developing rapidly. However, achieving Vision 2030’s goal of high‐quality care requires examining nursing autonomy to understand the role of nurses in advancing the healthcare system.

To illustrate the structural constraints that can shape nursing autonomy in hospital practice, decision‐making is often organised around physician authorisation pathways [2, 5]. In many settings, nursing care planning is closely aligned with the medical management plan, which can limit independent clinical judgement in treatment progression [3, 6]. Medication‐related decisions typically require medical orders, such as initiating, adjusting doses or selecting alternatives, and requests for laboratory or imaging investigations are commonly physician‐authorised even when nurses identify clinical needs [2]. Routine procedures (e.g., cannulation, catheterisation and vital sign monitoring) may also be governed by written orders and protocols rather than initiated solely through independent nursing assessment [2, 6]. Discharge decisions, follow‐up arrangements and treatment progression are similarly frequently contingent on medical authorisation [3, 5].

In Saudi Arabia, the urgency of examining nursing autonomy is intensified by the rapid pace of health system transformation under Vision 2030 and the increasing reliance on team‐based, high‐acuity hospital care. In referral hospital settings, constrained autonomy can have immediate implications for interprofessional decision‐making, patient advocacy, and workforce stability.

Professional nursing autonomy is a key to improving job performance and patient care. Increased autonomy is positively correlated with job satisfaction and work commitment among nurses. This, in turn, improves performance in critical care settings [7]. Consequently, creating autonomous environments is essential for providing optimal healthcare.

International evidence links professional autonomy to nurses’ clinical judgement, workforce outcomes, and quality of care [2]. In Saudi Arabia, Alruwaili and Abuadas [8] reported autonomy scores on a 1–5 scale and interpreted the overall level as moderate, with higher autonomy in patient‐care decisions than in unit operations.

In the past, nurses were regarded as playing a supportive role to physicians. However, nursing has evolved into an independent profession, with standards of practice, expertise, and educational pathways [6]. Additionally, autonomy enables nurses to use their clinical judgement, start treatments, and advocate for the best interests of their patients [5]. Identifying the facilitators and barriers to autonomy in Saudi Arabia is vital for empowering nurses and increasing their contribution to the healthcare system.

In nursing, professional autonomy refers to nurses’ capacity and authority to make accountable, evidence‐informed decisions regarding patient care within their defined scope of practice [9]. Commonly described dimensions include clinical autonomy, encompassing both independent and collaborative clinical judgement; control over nursing practice, reflecting nurses’ influence over practice standards and policies; and work autonomy, referring to unit‐level discretion in structuring and sequencing care delivery [1]. These dimensions informed the development of the interview guide as sensitising concepts; however, analysis and coding were conducted inductively and were not constrained by a prespecified dimensional framework.

Nurses are empowered when their autonomy is recognised and respected, which improves patient care and advances the profession as a whole. Higher levels of authority are linked to greater job satisfaction, better patient care and general improvements throughout the healthcare system [9].

While existing research in Saudi Arabia and comparable settings has largely quantified nursing autonomy using survey measures, it offers limited insight into how autonomy is lived, negotiated and constrained in everyday hospital practice. A qualitative descriptive approach is therefore needed to clarify the underlying organisational, relational, and cultural mechanisms that shape autonomy in practice, addressing this evidence gap.

### 1.1. Aim

This study aimed to explore how nurses experience and negotiate professional autonomy in Saudi hospital settings and to identify the organisational, relational and cultural factors that shape, enable or constrain autonomous practice.

### 1.2. Research Questions

1. How do nurses describe their experiences and negotiations of professional autonomy in Saudi hospital settings?
2. What organisational, relational, and cultural factors do nurses identify as shaping, facilitating, or constraining professional autonomy in everyday practice?

## 2. Materials and Methods

### 2.1. Study Design

A qualitative descriptive design was employed to capture participants’ perceptions and experiences of workplace barriers to nursing autonomy in their natural clinical context. Qualitative description is appropriate for studies aiming to capture naturally occurring phenomena without imposing theoretical or interpretive frameworks on participants’ accounts [10, 11]. The analysis was grounded in participants’ own words, thereby offering a contextualised understanding of how nurses experience and interpret autonomy in their daily clinical practice. This design generates findings that are meaningful to practitioners and transferable to existing healthcare settings. Consistent with the qualitative descriptive intent, the analytic approach operated primarily at a semantic level, staying close to participants’ explicit accounts; this is consistent with reflexive thematic analysis applied in its descriptive rather than latent or highly interpretive mode [12].

### 2.2. Study Setting

The study was conducted in two referral hospitals in northern Saudi Arabia. Hospital A has 450 beds, and Hospital B has 300 beds. Both hospitals serve as referral hubs for the city in which they are located, and they also receive referrals from nearby towns and surrounding areas, providing services consistent with their referral roles. In the context of Vision 2030 health system restructuring, both hospitals were affiliated with and supervised by a health cluster during the study period, which is relevant to transferability. The hospitals were purposively selected because their referral status and bed capacity provided an appropriate context for examining nursing autonomy within contemporary governance arrangements, consistent with recommendations to select sites that provide variation and contextual depth in qualitative research [13].

### 2.3. Study Sample

Eligible participants were registered nurses and nurse managers currently employed in the participating hospitals and with at least 3 years of clinical or managerial experience, ensuring familiarity with the professional, organisational, and ethical dimensions of nursing practice. Recruitment targeted a range of clinical and managerial areas to capture variation in autonomy experiences. Nationality was not used as an inclusion or exclusion criterion; however, all participants who consented and completed interviews were Saudi nurses, and all chose to be interviewed in Arabic. Nurses not employed in the participating hospitals during the recruitment period were excluded. A purposive sampling approach was used to recruit information‐rich participants who could provide in‐depth insights into the phenomenon of interest, namely, professional autonomy in nursing practice. Sample size was determined iteratively. Data collection and preliminary analysis proceeded concurrently, and recruitment continued until thematic sufficiency was reached, defined as the point at which subsequent interviews no longer contributed substantively new insights relevant to the study aim, and the themes were adequately developed across participants. Participants were recruited through a combination of face‐to‐face invitations and follow‐up emails. Initial invitations were shared by the researcher during scheduled departmental meetings, with further study information provided by email. To support wider dissemination, printed study information materials were distributed to potential participants by two nursing students who had no supervisory or evaluative authority over nursing staff, thereby posing no coercive risk to voluntary participation. To acknowledge potential residual power dynamics in group settings, no on‐the‐spot consent was sought during meetings; instead, nurses were invited to respond privately afterward (e.g., by email or direct contact). Participation was explicitly described as voluntary, with no consequences for declining, and nursing administrators were not directly involved in the selection or invitation process or informed of who accepted or declined, thereby minimising the risk of perceived coercion. Skår [3] notes that experienced nurses are better positioned to describe the complex features of autonomy, particularly its relationship with institutional norms and interprofessional dynamics. Their views were essential for capturing the facilitators and constraints that affect professional autonomy.

### 2.4. Data Collection

Semistructured interviews were used to gather detailed insights into how nurses perceived and experienced professional autonomy. The researcher developed a semistructured interview guide with open‐ended questions and prompts, grounded in the study’s aim and relevant literature on nursing autonomy. The guide explored participants’ experiences of autonomy in everyday practice, organisational and hierarchical influences on decision‐making, interactions with other health professionals, and workplace conditions perceived as enabling or constraining autonomous nursing practice. According to McIntosh and Morse [14], semistructured interviews blend standardisation with flexibility by combining guided questioning with participant‐led discussion, fostering a focused yet open‐ended conversation that facilitates in‐depth exploration of the phenomenon of nursing autonomy as experienced in practice.

The interviews were conducted by the researcher in Arabic to support natural, context‐sensitive accounts and to ensure that participants felt linguistically and culturally at ease. Interviews were transcribed verbatim in Arabic and then translated into English by the researcher, who is fluent in both languages. Formal forward–backward translation was not used; instead, translation was managed as a process of meaning equivalence. Translated transcripts were checked against the original Arabic audio recordings, and any ambiguous wording was revisited and refined prior to coding to ensure that participants’ intended meanings were retained. Probes were used to elicit concrete examples and to explore how autonomy was negotiated in routine clinical situations. The interviews were held face‐to‐face in private, quiet spaces within the hospital settings to encourage openness and ensure confidentiality. With participants’ informed consent, all interviews were audio recorded using a digital recorder to ensure accuracy and completeness.

Each participant was interviewed once. During interviews, the researcher recorded brief field notes to capture contextual cues and immediate impressions, which were expanded as needed to support ongoing reflection and analysis. Data collection and preliminary analysis were conducted iteratively and concurrently, with the researcher’s developing analytical observations informing subsequent probing and the exploration of areas of growing relevance in later interviews. Data collection continued until analytic sufficiency was reached, as judged by the researcher’s assessment that further interviews were unlikely to substantially deepen understanding of the phenomenon [15]. Each interview lasted approximately 1 h, consistent with qualitative best practices for generating sufficiently detailed narratives suitable for reflexive thematic analysis [16].

### 2.5. Data Analysis

Reflexive thematic analysis was employed to analyse the interview data, following the approach outlined by Braun and Clarke [12, 17]. Reflexive thematic analysis is particularly well‐suited to qualitative descriptive research, as it foregrounds the researcher’s active role in constructing meaning from data while remaining grounded in participants’ accounts [12]. The first stage of the analysis involved data familiarisation, including transcribing the interviews and reading the transcripts multiple times to develop a comprehensive understanding of what had been said. The researcher generated initial codes inductively by systematically labelling data segments related to nursing autonomy. These codes were then clustered into conceptually related categories and refined through iterative review to build an overarching thematic structure. Codes and categories were organised using qualitative data analysis software (NVivo 12) to support systematic retrieval and comparison across transcripts. Because the researcher served as the sole analyst, intercoder disagreement procedures were not applicable. Consistent with the qualitative descriptive intent, coding and theme development were undertaken primarily at a semantic level, staying close to participants’ explicit accounts rather than generating latent, highly interpretive codes. Any hierarchical grouping of codes was used as an organisational device rather than a prespecified codebook or fixed analytic framework. This structure helped clarify relationships among recurring ideas across the dataset and supported a coherent and analytically grounded account of workplace barriers to nursing autonomy.

To enhance the credibility of this analytic process, several verification strategies were integrated during and after theme development. As themes were constructed and developed, they were repeatedly refined to ensure coherence, relevance, and interpretive credibility. Member checking was conducted with a subset of participants (*n* = 6) to assess whether the constructed themes accurately reflected what they intended to convey in their interviews. Participants were invited to comment on the clarity and resonance of the preliminary thematic summary, and their feedback informed minor refinements to the wording and organisation of the themes. Following up with all participants was not feasible due to clinical workload and scheduling constraints. To enhance reflexivity, the researcher maintained an audit trail and wrote self‐reflective memos throughout the analysis. Reflexive memoing also addressed the researcher’s position as a Saudi nurse and academic, documenting how insider familiarity and analytic distance were considered in interpretation. For instance, memos captured the researcher’s initial assumptions and reactions to specific accounts and recorded the rationale for analytic decisions during theme development (e.g., why certain overlapping codes were merged, renamed or retained to better reflect participants’ intended meanings). These strategies contributed to the trustworthiness and integrity of the findings [13]. Although the study was conducted independently, the researcher occasionally engaged in informal discussions with academic peers about emerging ideas that challenged assumptions and enhanced analytical reflexivity. These discussions were brief debriefing conversations focused on assessing the theme clarity and considering alternative interpretations. Themes and subthemes were organised to summarise patterned meanings in participants’ accounts and to support a descriptive presentation of workplace barriers to nursing autonomy.

### 2.6. Ethical Considerations

Before data collection commenced, ethical approval was obtained from the Northern Border University bioethics committee (HAP‐09‐A‐03). Ethical rigour was key to the methodology, and participants’ rights, privacy, dignity, and confidentiality were safeguarded. All participants were informed of the aim, methodology, potential risks, and benefits of the study so that they could provide informed consent. The participants were invited to ask questions to ensure that they were fully informed, and they freely volunteered to participate in the study. Thus, informed consent was obtained from all of them. To manage potential distress, participants were reminded that they could decline to answer any question, pause the interview, or stop participation at any time without providing a reason; where discomfort was observed or expressed, the interview was slowed or redirected according to participant preference.

Personal identifiers were not included in the transcripts, and the latter were stored on password‐protected devices accessible solely to the research team. Data were anonymised, and participants were informed that they could leave the study at any point and that this would not impact their employment status or access to healthcare services [13]. Given the potential for deductive identification in small departments, quotations were screened to remove or generalise potentially identifying contextual details (e.g., unit names), and participants were identified using numeric codes (e.g., P1–P24) rather than their names. To minimise power imbalances in a workplace setting, participation was explicitly framed as voluntary and confidential, and nursing administrators were neither involved in recruitment nor informed of who participated.

## 3. Results

The study was based on data from 24 participants, comprising staff nurses and nurse managers from two referral hospitals in northern Saudi Arabia. The group represented a range of professional roles and areas of expertise, thereby offering a broad view of nursing autonomy. Most participants were female and had an average of 7 years of professional experience. Table 1 summarises the participants’ demographic characteristics.

**TABLE 1:** Demographic characteristics of participants.

Participant characteristics	Details
Total Participants	24

Study Context	Two referral hospitals

Sex Breakdown	19 females (79%)
	5 males (21%)

Average Experience (years)	7 years

Age (years)	25–30: 6 (25%)
	31–35: 11 (46%)
	36–40: 5 (21%)
	41–46: 2 (8%)

Nationality	Saudi: 24 (100%)

Level of Education	Diploma: 3 (13%)
	Bachelor’s: 19 (79%)
	Master’s: 2 (8%)

Professional Roles	16 Staff Nurses (67%)
	8 Nurse Managers (33%)

Areas of Expertise	Community Health, Obstetrics, Critical Care, Medical‐Surgical Nursing

### 3.1. Presentation of Themes and Subthemes

Data analysis highlighted three linked themes illustrating how nurses at two referral hospitals in northern Saudi Arabia view and practise professional autonomy. The themes comprised structured dependency, which investigates the impact of organisational hierarchies and restricted managerial influence; navigating interprofessional dynamics, which examines the tensions and collaboration found within interdisciplinary teams; and communication and professional identity, which explores how nurses express autonomy through role assertion and interaction. Each of these themes is supported by personal accounts and was verified using evidence from the literature.

#### 3.1.1. Theme 1: Structured Dependency

This theme mirrors the hierarchical structure and top‐down decision‐making, which participants frequently referred to as a hurdle to exercising professional autonomy. Nurses felt that their clinical environment was characterised by inflexible institutional control and that their opinions were regularly overlooked while bureaucratic and managerial directives were prioritised.

##### 3.1.1.1. Subtheme 1.1: Organisational Hierarchy

A number of nurses stated that their clinical judgement was often overshadowed by administrative decisions or policies. Even when they were confident in the accuracy of their assessment or intervention strategy, they were obliged to follow instructions without questioning their appropriateness:

“There is an expectation that we would just follow the directive, whether we agreed with it or not.” (P13)

This subtheme reflects constraints on formal authority and decision‐making rights, with decision‐making legitimacy perceived as residing above the nursing role. This perception of marginalisation led to feelings of disempowerment and, occasionally, professional disengagement. Such experiences bolster broader criticism of a centralised decision‐making process that limits the extent to which nurses participate in shared governance.

##### 3.1.1.2. Subtheme 1.2: Insufficient Influence of Nurse Managers

Whereas Subtheme 1.1 focuses on formal decision authority within hierarchical structures, this subtheme addresses nurse managers’ constrained capacity to advocate for their staff and to foster the psychological safety needed for speaking up. Participants consistently described leadership structures as actively limiting managers’ ability to advocate effectively on behalf of their teams, reinforcing a broader sense of diminished professional authority. As one participant stated:

“Without the support of our supervisor, it is hard to voice our independence.” (P4) “I do not want to blame my colleagues in management, but they need to show more courage and act in the collective interest of nurses.” (P13)

A nurse manager also described feeling that their input was not taken seriously, reinforcing constrained advocacy and limited voice within managerial channels:

“…. you know, sometimes I feel that my opinion has not been taken seriously, and I never felt that upper management, or even fellow nurse managers, valued it.” (P13)

These comments reflect a leadership style in which administrative compliance rather than strategic influences or mentoring defines managerial roles. They also demonstrate ingrained hierarchical dependency.

#### 3.1.2. Theme 2: Navigating Interprofessional Dynamics

This theme examines how relationships and interactions with physicians and multidisciplinary teams affect autonomy. Participants spoke of different degrees of interprofessional cooperation, and most positive interactions were reported when dealing with younger or more collaborative physicians. Nevertheless, overall, the most common experience was one where nurses played a subordinate role and had limited influence. Interprofessional interaction was frequently described as characterised by directional authority, where decision‐making tended to be physician‐led rather than jointly negotiated.

##### 3.1.2.1. Subtheme 2.1: Nurse–Physician Collaboration

A number of nurses reported instances of effective collaboration with junior medical staff:

“(smiling briefly) …I do have good cooperation with some of the younger doctors, and we understand each other well. (pauses, then sighs) But that is not how it is with most of the senior physicians I have worked with—I am just expected to take orders and follow them…” (P8) “(slightly frustrated tone) …doctors are seen as more authoritative. That affects how much input we have…” (P12) “…sometimes collaboration feels one‐sided—doctors lead, and we follow…” (P8)

This lack of equal status frequently makes nurses feel marginalised and excluded from critical decisions. One participant stated, “…patients are not well advocated for when the doctor ignores our viewpoint…” (P17)

These accounts reflect a directional collaboration and unidirectional decision‐making, in which nurses’ contributions were described as contingent on physician receptiveness rather than routinely embedded in team processes.

This imbalance in decision‐making authority underscores how professional hierarchies remain entrenched in the system. It undermines respect within interdisciplinary teams and the focus on patient‐centred care.

##### 3.1.2.2. Subtheme 2.2: Role Ambiguity and Conflict

The lack of clarity when it comes to sharing responsibilities between nurses and other healthcare workers increases stress and causes confusion. Certain participants questioned their scope of practice in relation to others:

“Sometimes, as a nurse technician, I am unsure which tasks are mine versus those of a staff nurse.” (P11) “(shrugs) …I believe that overlapping roles cause confusion and delays in decisions regarding patient care…” (P17) “Even with job descriptions, responsibilities are often blurred, leading to conflict.” (P11)

This lack of clarity regarding individual roles and responsibilities reduces nurses’ sense of ownership over their clinical responsibilities and can disrupt teamwork.

#### 3.1.3. Theme 3: Communication and Professional Identity

This theme examines how nurses navigate interpersonal dynamics and use communication strategies to assert their professional role. Participants asserted that their speech, demeanour and confidence contributed to whether colleagues listened to them or afforded them professional respect.

##### 3.1.3.1. Subtheme 3.1: Norms of Professional Communication

Many participants stated that they were wary of freely expressing themselves, particularly when interacting with authority figures:

“In our setting, respect for authority is very important; therefore, challenging decisions directly can be difficult at times.” (P2) “(sighs) …you feel hesitant to speak up because questioning superiors can be seen as disrespectful…” (P8)

This self‐censorship, albeit culturally influenced, reflects power dynamics that can often undermine nurses’ professional assertiveness.

##### 3.1.3.2. Subtheme 3.2: Role Assertion in Diverse Work Environments

Other nurses recounted their strategies to gain credibility and recognition during teamwork. These included modulating their tone of voice, building rapport with their colleagues, and demonstrating their competence by consistently performing well in the clinics.

“Sometimes I have to change how I say things so that people actually listen to what I mean.” (P8) “At the beginning, a few people appeared surprised to see me working here, but after a while, they realised that I take my job seriously.” (P2)

These accounts highlight the relational and interactional nature of professional identity, with autonomy described as being negotiated through communication, credibility, and demonstrated competence in everyday practice.

The findings show that professional autonomy is a robust, contextually embedded phenomenon. Rather than being viewed as a standardised or solely institutional trait, autonomy develops through the daily interactions between policy, team relationships, leadership, and individual voices. Consequently, any initiative aimed at strengthening nursing autonomy should address both systemic structures and the interactions that shape practice at every level of the healthcare system.

Overall, autonomy was uneven across practice domains. Low autonomy predominated in situations shaped by top‐down decision‐making and hierarchical control, where nurses’ independent judgement was routinely subordinated to organisational and professional authority. In contrast, higher independence was observed in limited circumstances—particularly when interprofessional relationships were collaborative (e.g., with more receptive physicians) and when nurses strategically asserted their role through communication and demonstrated competence. These instances of greater autonomy were episodic and relational, rather than consistently embedded in organisational structures.

### 3.2. Limitations

This study has notable limitations. First, the findings may not be transferable to other healthcare contexts, private institutions, or hospitals operating under different health‐cluster conditions, as the study were conducted in two referral hospitals in the northern region of the country. The Saudi healthcare system is currently shifting away from General Directorates of Health Affairs towards regionally governed health clusters, which now have full operational responsibility for all their hospitals and healthcare centers, including management, staffing and service delivery. As a result, variations are likely to arise in how the clusters implement national strategies. This, in turn, could affect nurses’ experiences of autonomy across different settings. Both hospitals were supervised by a health cluster during the study period; therefore, transferability may be more limited in settings with different governance arrangements, operational conditions, organisational cultures or resource profiles. The participants in this study represent a broad range of roles and specialties; however, their experiences may not completely reflect the diversity of nursing practice throughout Saudi Arabia.

Second, as is the case with all qualitative studies, the findings are shaped by the researchers’ interpretations and the context in which data were gathered. In this study, analysis was conducted by a single researcher, which may introduce residual interpretive bias in coding decisions and theme boundaries, despite reflexive memoing and peer debriefing. To strengthen the study’s credibility and trustworthiness, reflexive journals were maintained throughout the research process. These enabled the researcher to critically evaluate assumptions and positionality at several stages of data collection and analysis. In addition, because interviews addressed autonomy within hierarchical workplaces, participants may have moderated criticism or framed responses in socially desirable ways, despite assurances of confidentiality and voluntariness. Practical obstacles also shaped the study process: clinical workload and shift patterns limited opportunities for re‐contact, which restricted member checking to a subset of participants, and the hospital setting introduced the possibility of time pressures or interruptions that could influence the depth of disclosure in some interviews.

Another limitation stems from the evolving nature of national initiatives, such as the SCFHS postgraduate diploma programmes. These may have longer‐term effects on autonomy, which could not be assessed during the data collection period of this study. Future researchers could examine the longitudinal impact of these types of policy interventions and systematically investigate how advanced practice roles develop over time.

Despite these limitations, this study contributes positively to the debate on nursing autonomy within the Saudi healthcare system. The study highlights major barriers to autonomy in leadership, effective collaboration, and organisational culture and identifies prospective transformation points. The study findings emphasise the complexity of the concept of autonomy as a structural and interpersonal construct shaped by policy, communication, and professional identity.

## 4. Discussion

This study makes a valuable contribution to the expanding body of qualitative research on nursing autonomy, offering insights drawn from the experiences of nurses working in two referral hospitals in the north of Saudi Arabia. The participants’ narratives describe autonomy as a dynamic process, rather than role‐based privilege or a static trait, which is shaped by an interlocking pattern of organisational structures, cultural expectations, and professional relationships. The findings illustrate the ongoing negotiation between professional desires and limitations stemming from the system, highlighting how autonomy is both restricted and enacted in day‐to‐day practice [2].

The structured dependency theme reveals how hierarchical control, particularly in decision‐making and policy compliance, continues to frame the work of the nursing staff. Most participants stated that they had little, if any, influence over institutional processes, even in situations where their clinical expertise could significantly contribute to decisions about patient care. This aligns with earlier evidence suggesting that command‐based structures in healthcare limit nurses’ professional agency and contribute to moral distress [2, 4]. Nurses working in such environments frequently experience burnout and diminished motivation, partly due to bureaucratic constraints and centralised leadership cultures that restrict autonomous practice [4, 7]. However, the findings indicate that dependency‐oriented practice is not solely a product of formal organisational structures, such as hierarchical decision‐making processes and governance arrangements, but is equally shaped by managerial cultures that prioritise compliance over professional collaboration, as reflected in the limited authority afforded to nurse managers. In this sense, structural constraints and workplace culture appear to operate as mutually reinforcing conditions rather than discrete or competing explanations for restricted nursing autonomy. Cummings et al. [4] suggested that this calls for leadership models that are based on distributed authority and clinical decision‐making in the workplace. Similar findings were reported in earlier studies in Saudi healthcare settings, where nurse managers reported limited authority and influence within centralised decision‐making structures [18].

Survey‐based work in Saudi settings has described autonomy levels as moderate, often indicating that autonomy may be greater in direct patient‐care decisions than in unit‐level operational decisions [8]. The present qualitative findings do not contradict these estimates; rather, they help explain how ‘moderate’ autonomy can coexist with pronounced day‐to‐day dependency when decision authority remains concentrated hierarchically, and nurses’ influence is contingent on relational and managerial conditions. In this sense, autonomy appears less as a stable individual attribute and more as context‐dependent and negotiated, which is consistent with international qualitative accounts that locate autonomy within organisational and interprofessional conditions [2, 3].

International literature similarly identifies organisational hierarchy and leadership practices as conditions that can constrain nurses’ autonomy and voice within healthcare organisations [2, 4]. In the Saudi context, the findings suggest these structural constraints are experienced through particularly pronounced top‐down decision‐making, which may intensify day‐to‐day dependency and limit nurses’ influence over clinical and organisational decisions.

When discussing navigating interprofessional dynamics, nurses described how their relationships with physicians varied according to the individual. They also emphasised the importance of role clarity and mutual respect. Most participants expressed that established hierarchies tended to marginalise their input, although younger doctors were often more collaborative. These findings echo earlier studies on interprofessional power differentials and communication barriers, which limit effective teamwork and reduce nurses’ efforts to advocate for their patients [6]. The literature has established that equitable collaboration between nurses and physicians improves safe care and raises nurses’ job satisfaction [19]. Furthermore, role ambiguity, in particular between staff nurses and technicians, was reported to be a source of inefficiency and frustration. Clearly, autonomy was reduced when roles became unclear. Havaei et al. [20] found that role confusion between nursing staff of differing classifications was associated with compromised perceptions of care quality and increased patient adverse events. Similar power differentials between nurses and physicians, affecting nurses’ influence in team decision‐making, have also been described in other settings [6, 21].

The communication and professional identity theme focuses on how autonomy is manifested in the moment, through interaction, demeanour, and language. Nurses described how they endeavoured to gain respect by modifying their behaviour and tone of voice so that they would be heard. This was particularly true in hierarchical or male‐dominated environments. These strategies are an overlooked emotional approach that nurses harness to assert their credibility and better advocate for the patients in their care. This pattern can also be understood through the lens of voice and silence in clinical teams, where speaking up is shaped by perceived interpersonal risk and expectations about how concerns will be received [22]. Psychological safety is a relevant explanatory construct here, referring to a team climate in which staff feel able to raise concerns, question decisions, and contribute ideas without fear of negative consequences [23]. Viewed in this way, participants’ communication strategies reflect attempts to create conditions for voice and credibility in settings where psychological safety may be uneven. The emotional and communicative work inherent in nursing has been thoroughly documented and is often vital in settings where nurses are not automatically granted professional authority [24]. Furthermore, the findings support arguments that professional autonomy involves not only clinical judgement but also the ability to negotiate status within a team and interpersonal confidence.

### 4.1. Implications for Nursing Management and Policy

Implications are presented for nursing management and policy, focusing on leadership practices, shared governance, and institutional policies that shape nurses’ decision‐making authority. These implications are presented at two levels: first, practice and management implications are drawn directly from the findings in two referral hospitals and reflect participants’ reported constraints and enabling conditions for autonomy; second, broader policy recommendations are offered as considerations informed by wider evidence and national reform priorities and should be interpreted in light of the study’s context‐bound scope. The findings of this study align with the objectives of Saudi Arabia’s Vision 2030, particularly the Health Sector Transformation Program, designed to improve the quality and efficiency of healthcare services by empowering its workforce. Nursing autonomy is a key element of that vision. Driving greater professional independence, upgrading interprofessional collaboration, and investing in nursing leadership will increase patient satisfaction, promote innovation, and create a sustainable healthcare system, in line with the goals of Vision 2030.

The Saudi Commission for Health Specialties recently launched a 1‐year postgraduate diploma in critical care, emergency nursing, and other specialties. Although such programmes are a meaningful advance in specialised nursing roles, it remains unclear whether they will bolster nurses’ clinical autonomy. For this type of training to be empowering, rather than merely procedural, changing institutional cultures and clarifying roles and decision‐making authority in the workplace are essential. However, these initiatives do offer a foundation for developing advanced practice roles, such as nurse practitioners. This would, in turn, allow nurses to expand their expertise and make significant contributions to healthcare leadership and patient care.

Vision 2030 calls for developing healthcare professionals who can drive improvement by focusing on accountability, continuous learning, and shared governance. These principles are fundamental to the concept of autonomy.

Introducing master’s‐ and doctoral‐level clinical nursing degrees, such as advanced practice nursing programmes modeled on those offered in the United States, the United Kingdom and Canada, is promising and may be considered as a longer‐term strategy, informed by broader international evidence, to advance clinical autonomy in Saudi Arabia. Evidence suggests that advanced practice roles, such as nurse practitioners, clinical nurse specialists and nurse consultants, contribute to higher‐quality care, improved clinical decision‐making and greater workforce retention through enhanced autonomy and leadership engagement [25, 26]. Here, aligning regulatory frameworks, clinical privileges and educational pathways means ensuring that scope‐of‐practice regulations and professional licensure authorise advanced roles; that hospital credentialing processes grant corresponding clinical privileges, such as ordering diagnostic tests, prescribing within agreed formularies and leading advanced clinical decision‐making; and that accredited postgraduate education and competency assessment prepare nurses to meet these role requirements.

The findings of this study have numerous implications for nursing practice, leadership, education and policy. Promoting autonomy in practice will require efforts to reduce rigid bureaucracy in organisations and enable nurses to exercise discretion in clinical decision‐making settings. Introducing shared governance or interdisciplinary councils will provide platforms where nurses can offer their input and share accountability.

In terms of leadership, nurse managers and administrators must model and support autonomy by creating psychologically safe workplaces, advocating for their colleagues, and promoting reflective discussions. These initiatives are relevant in the context of Saudi Arabia, as earlier research identified leadership gaps and hierarchical systems that restrict nursing practice [27]. Inclusive leadership styles have been associated with improved nurse perceptions of autonomy and professional engagement, partly through the psychological safety and organisational support they generate [4, 28, 29].

Nursing curricula should be revised to include modules on communication, ethical reasoning, systems thinking, and advocacy. Embedding autonomy into professional identity development at an early stage may prepare graduates to be assertive and responsible in their subsequent practice in the increasingly complex health setting.

At the policy level, healthcare institutions and regulatory bodies must ensure that policies align with empowerment goals by formally protecting the decision‐making authority of nurses. Systemic barriers to autonomy can be dismantled by policies which support role clarity, continuous professional development, and collaboration across professions.

Nonetheless, these implications should be realised with cultural sensitivity. Where respect for hierarchy and authority form part of cultural values, promoting autonomy must be presented as constructive engagement rather than a challenge or form of rejection. This can be achieved through shared leadership, respectful discussions, and collaborative problem‐solving.

While these recommendations are informed by the Saudi context and based on two referral hospitals, the underlying issues they address (hierarchical decision‐making, role clarity, and conditions for professional voice) may be relevant to other settings; however, transferability should be approached cautiously given contextual differences. These recommendations, albeit presented in the context of Saudi Arabia, apply to health systems worldwide. Healthcare is becoming increasingly collaborative, complex, and culturally diverse. Thus, supporting nursing autonomy is vital for ensuring safe, effective, and patient‐centred care.

## 5. Conclusions

Nursing autonomy plays a pivotal, yet variably supported role, in professional practice in Saudi healthcare. This study demonstrates how nurses continue to navigate entrenched structural and cultural influences that affect their ability to make independent decisions and to make meaningful contributions to patient care. Nevertheless, the tide of policy reforms, evolving educational programs, and a growing focus on workforce development in Saudi Arabia provide opportunities for change.

Autonomy will flourish if reforms extend beyond procedural training and address leadership abilities, interprofessional respect, and institutional responsiveness. To meet the goals of Vision 2030, culturally sensitive strategies that recognise the values of respect, dialogue, and collaboration must be embraced. Nurses should be equipped with technical expertise, as well as be empowered to use their clinical judgement confidently, with the knowledge that they will be supported.

Healthcare leaders who listen to nurses and acknowledge their experiences will be able to build environments that promote autonomy, raise retention rates, and improve the quality of patient care. In doing so, they will be meeting and translating the aims of Vision 2030 and building a strong and sustainable healthcare system for future generations.

Therefore, future research should explore how specific leadership styles, interprofessional dynamics, and organisational policies either foster or hinder nursing autonomy across different healthcare settings. Comparative studies across regions or healthcare sectors within Saudi Arabia could also offer valuable insights into how cultural and institutional contexts shape the experience of autonomy. Understanding these dimensions will be critical for designing strategies that genuinely empower nurses and strengthen the Saudi healthcare system.

## Author Contributions

Author Alenezi Ibrahim Naif was responsible for conceptualisation, methodology, data curation, writing–original draft preparation, validation, and reviewing and editing.

## Funding

This work was supported by the Deanship of Scientific Research at Northern Border University, Arar, Saudi Arabia (grant number NBU‐FPEJ‐2026‐1626‐01).

## Conflicts of Interest

The author declares no conflicts of interest.

## Data Availability

The data that support the findings of this study are available on request from the corresponding author. The data are not publicly available due to privacy or ethical restrictions.
